# Case Report: Extracorporeal Membrane Oxgenation for Rapidly Progressive Interstitial Lung Disease Associated With Clinically Amyopathic Dermatomyositis in a Post-partum Woman

**DOI:** 10.3389/fmed.2021.742823

**Published:** 2021-10-01

**Authors:** Qiao Gu, MengYuan Diao, Wei Hu, Man Huang, Ying Zhu

**Affiliations:** ^1^Department of Critical Care Medicine, Affiliated Hangzhou First People's Hospital, Zhejiang University School of Medicine, Hangzhou, China; ^2^Department of General Intensive Care Unit, Second Affiliated Hospital, Zhejiang University School of Medicine, Hangzhou, China

**Keywords:** amyopathic dermatomyositis, extracorporeal membrane oxygenation (ECMO), interstitial lung disease (ILD), lung transplantation, posterior reversible encephalopathy syndrome (PRES), case report

## Abstract

**Background:** Clinically amyopathic dermatomyositis (CADM) presented with rapid progressive interstitial lung disease (RP-ILD) is rare. Here, we present a case of a post-partum female with CADM complicated by severe RP-ILD managed with venovenous extracorporeal membrane oxygenation (V-V ECMO).

**Case Summary:** A 36-year-old woman was referred to a local hospital with cough and fever. She had a history of facial erythema and cough since an induction of labor for a stillborn fetus 2 months ago. Her status developed into RP-ILD with mediastinal emphysema and subcutaneous emphysema after admission, and V-V ECMO was initiated. After several failed attempts to wean the patient from ECMO, a decision was made to place the patient on the lung transplant waitlist. She underwent a double lung transplant on ECMO day 31 and received tacrolimus as an immunosuppressive regimen. The patient presented with positive anti-MDA5 and anti-Ro-52 antibodies and a high ferritin level, all of which indicated the presence of clinically amyopathic dermatomyositis (CADM). The patient was weaned from ECMO at 3 days after transplantation, but the patient's state of consciousness deteriorated, and head CT was considered for posterior reversible encephalopathy syndrome (PRES). After the temporary cessation of calcineurin inhibitors and a dosage reduction, the patient's state of consciousness returned to normal. Because of another disturbance of consciousness, the patient declined further treatment and was discharged 14 days after transplantation.

**Conclusion:** Early recognition of CADM can effectively improve patients' prognosis. ECMO should be considered as a supportive therapy in patients in acute respiratory failure secondary to RP-ILD.

## Introduction

Clinically amyopathic dermatomyositis (CADM) is defined as the presence of typical cutaneous manifestations of dermatomyositis (DM) along with absent or minimal muscle weakness in DM (1). Population-based data suggest that CADM occurs in ~20% of all adult DM cases (2), and the incidence of CADM is estimated to be 2.08 per 1 million persons (3). Patients with CADM have an increased risk of interstitial lung disease (ILD), and especially severe cases are complicated by life-threatening rapid progressive ILD (RP-ILD) (4), emphasizing the necessity and significance of early recognition and management of the disease. ILD more frequently occurs in patients with CADM and positive anti-melanoma differentiation-associated gene 5 (anti-MDA5) antibody, and these patients are more likely to progress to RP-ILD and refractory acute respiratory failure and often have a poorer prognosis than those with negative anti-MDA5 antibody (5).

For RP-ILD patients with critical hypoxemia or respiratory acidosis despite conventional therapies, particularly that accompanied by other respiratory complications, venovenous extracorporeal membrane oxygenation (VV-ECMO) may be a perfect choice. The treatment is sometimes a bridge to recovery, but limited options exist for CADM patients with refractory hypoxemia who fail to wean from ECMO (6). However, with no lung transplant consideration, using ECMO in such cases has been felt to be “bridge to nowhere” (7) due to the limited treatment response and overall options. The immunosuppressants used after transplants inevitably introduce complications; posterior reversible encephalopathy syndrome (PRES) is a common complication that manifests as various neurological symptoms (8). Tacrolimus, an important immunosuppressive drug for organ transplantation patients, is a major risk factor for PRES (9).

Here, we present the case of a post-partum female with CADM complicated by severe RP-ILD managed with VV-ECMO. Due to refractory respiratory failure, bilateral lung transplantation was eventually performed, but the patient ultimately developed posterior reversible encephalopathy syndrome.

## Case Presentation

**Figure 1:**
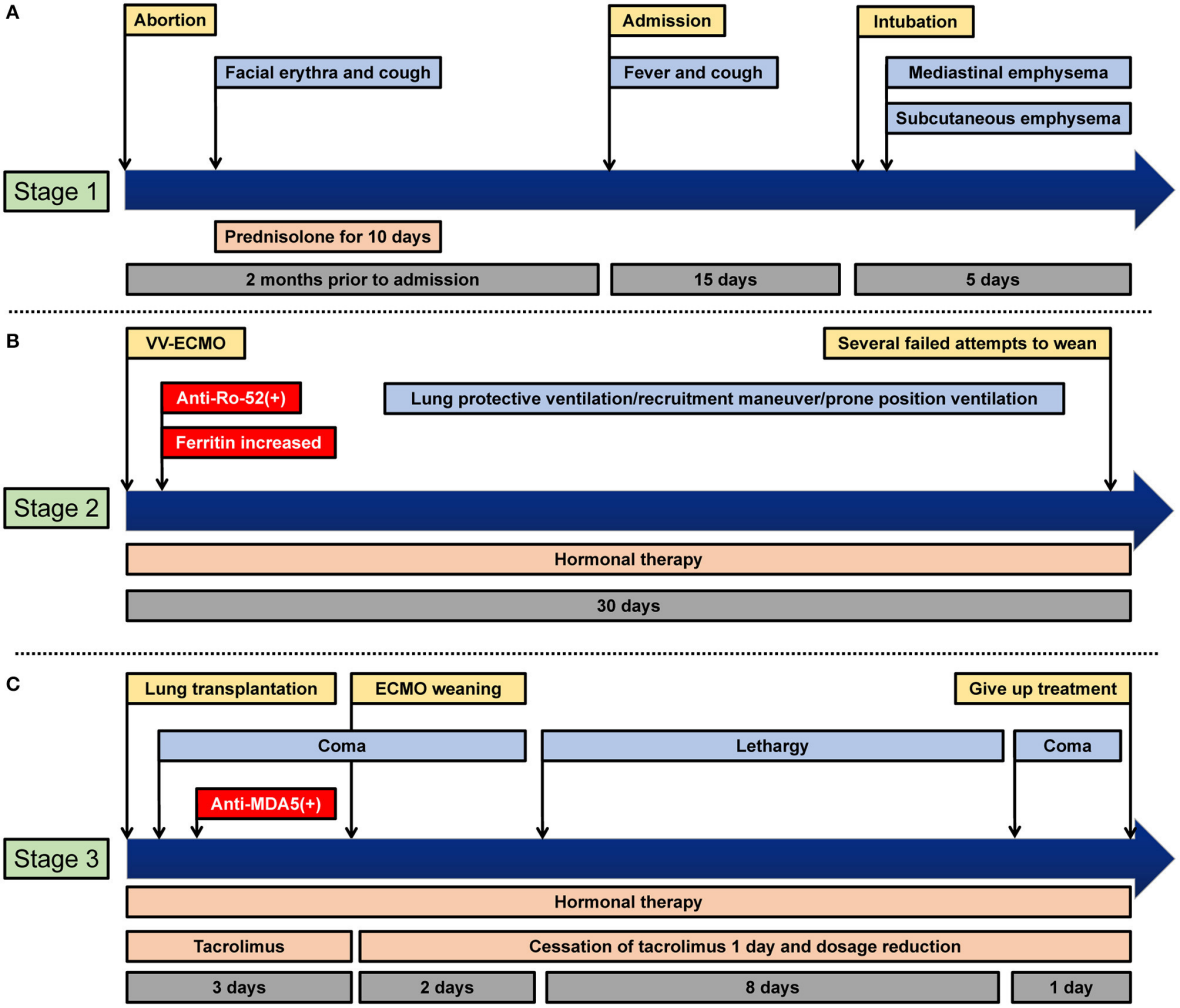
Clinical course of the patient. **(A)** Stage 1: from pathogeny to intubation. **(B)** Stage 2: ECMO stage. **(C)** Stage 3: transplant and post-transplant stages.

A 36-year-old previously healthy female visited a local hospital presenting with cough and fever. She had a history of spontaneous abortion twice and underwent induction of labor for a stillborn fetus 2 months before. Subsequently erythema on her face was noticed, accompanied by continuous cough, but no phlegm. Allergic disease was considered, and anti-allergic treatment was given, but her symptoms did not improve. Her condition was then managed with oral prednisolone for 10 days, and the facial erythema and cough disappeared. The patient's clinical course is shown in Figure 1.

**Figure 2:**
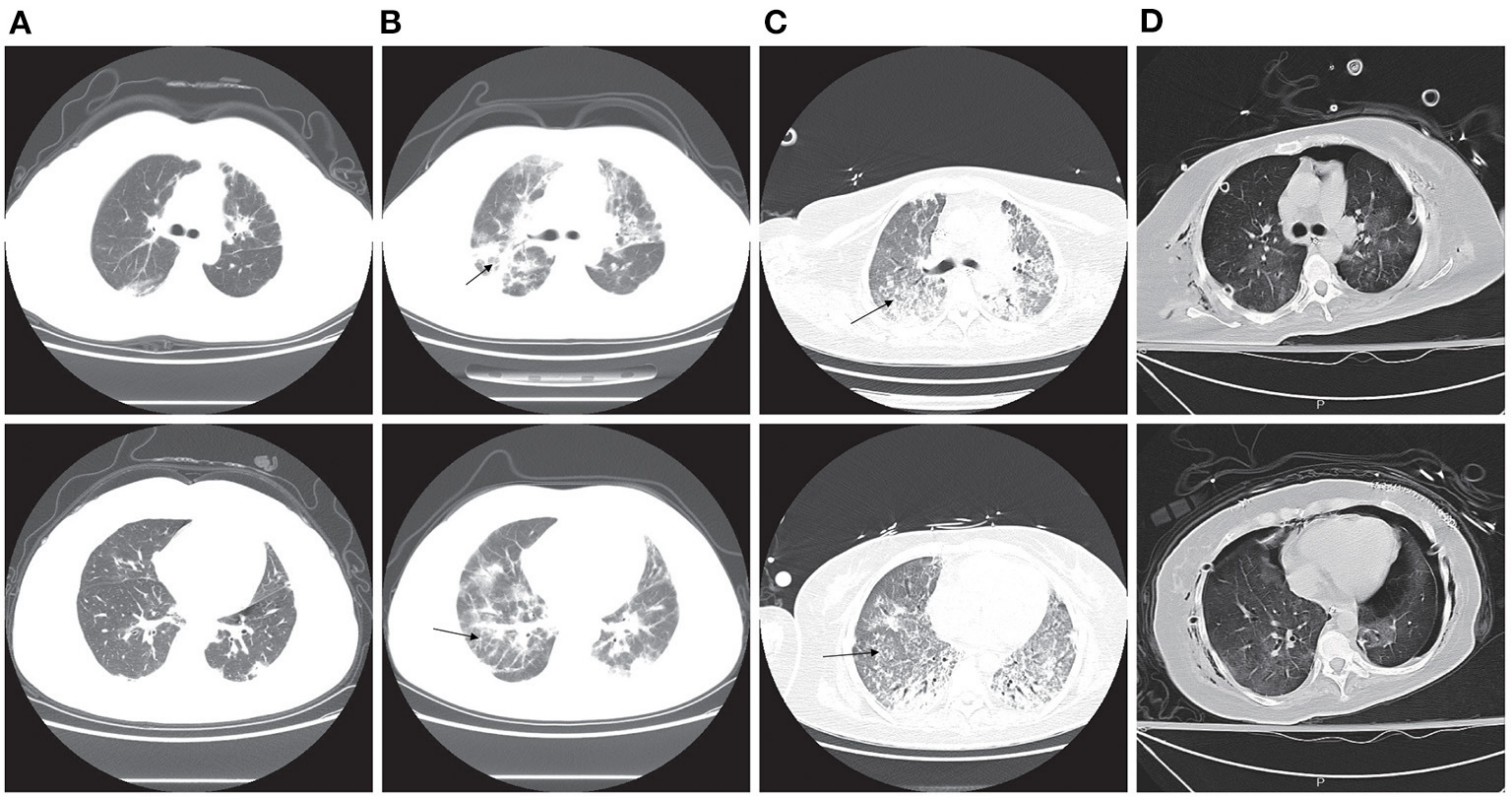
Computed tomography (CT) image of the chest. **(A)** CT of the chest shows bilateral ground glass opacities 9 days before intubation. **(B)** CT of the chest shows bilateral pulmonary patchy infiltrates, which was considered as interstitial pneumonia 3 days before intubation (arrowheads). **(C)** CT of the chest shows bilateral pulmonary extensive infiltrates and lobular interstitium thickness, which was considered as pulmonary fibrosis 23 days after ECMO (arrowheads). **(D)** CT of the chest showed bilateral pulmonary scattered infiltration 3 days after lung transplantation.

On admission, the patient showed no cutaneous and muscular manifestations. Computed tomography (CT) of the chest showed bilateral ground glass opacities (Figure 2A). Anti-MDA5 antibody was not measured because there was no consideration of CAMD and ILD. After receiving antibiotic therapy for 12 days, the patient's status did not improve and worsened in later stages; the clinical manifestations were shortness of breath and dyspnea. CT of the chest showed bilateral pulmonary patchy infiltrates, and interstitial pneumonia was considered (Figure 2B). Shortly thereafter, the patient presented with acute hypoxemic respiratory failure (PaO_2_/FiO_2_: 68 mmHg), and intubation and mechanical ventilation were subsequently performed 3 days after admission. Using next-generation sequencing (NGS) of the bronchoalveolar lavage fluid (BLF) sample and cultured isolates from the patients, *Pseudomonas aeruginosa, Stenotrophomonas maltophilia*, and *Pneumocystis jirovecii* were found. Sulfamethoxazole (SMZ) was added for the treatment of pneumocystis pneumonia (PCP) caused by *Pneumocystis jirovecii*. The patient' s respiratory status continued to deteriorate, and mediastinal emphysema and subcutaneous emphysema developed 4 days after invasive ventilation.

**Figure 3:**
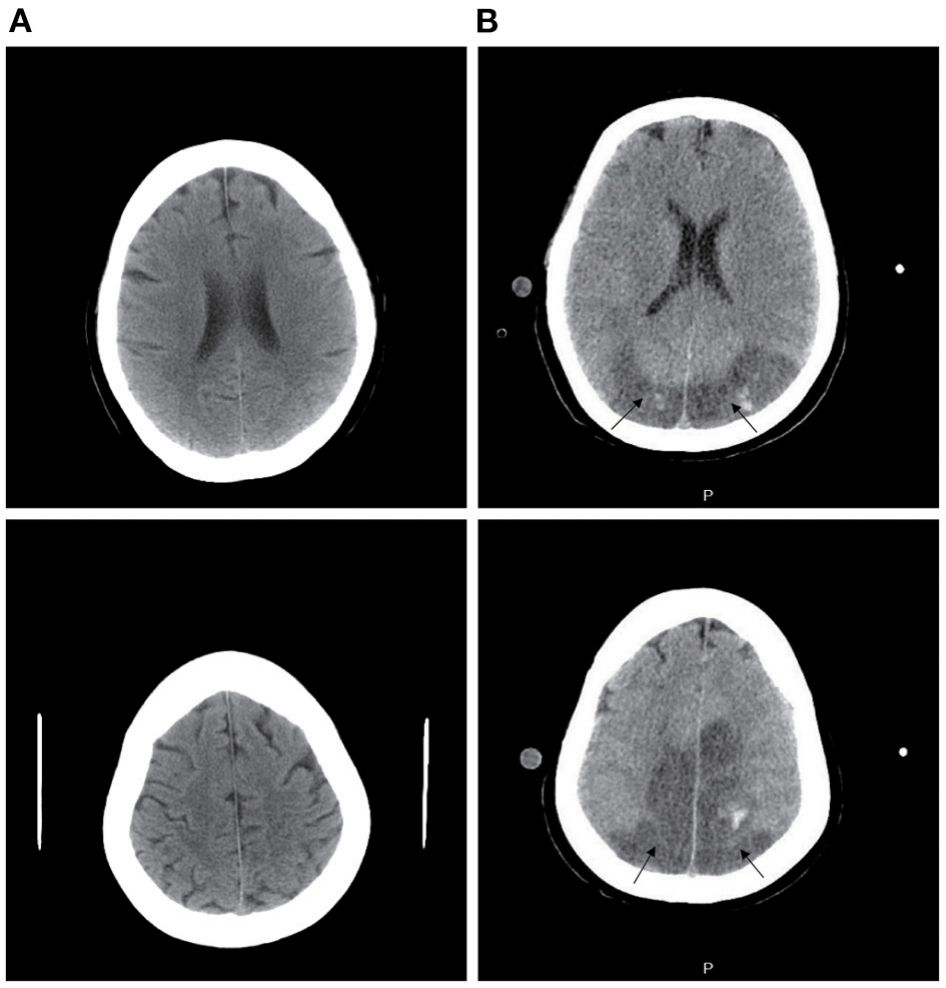
Computed tomography (CT) image of the head. **(A)** CT of the head is normal at the ECMO stage. **(B)** CT of the head shows bilateral parieto-occipital low-density lesions at day 3 after transplantation (arrowheads).

VV-ECMO *via* the right internal jugular and right femoral vein cannulation was initiated on ventilator day 4, and she was then referred to the ECMO center. Laboratory investigations revealed that serum anti-Ro-52 was positive via ELISA, and serum ferritin (SF) was significantly higher without elevated serum muscle enzymes. NGS of BLF also showed *Pneumocystis jirovecii* and *Acinetobacter baumannii*; thus, antibiotic therapy and SMZ were continued. Considering the COVID-19 epidemic in China, the patient received a nucleic acid test for COVID-19, but the result was negative. CT of the chest showed bilateral pulmonary extensive infiltrates and lobular interstitium thickness, and pulmonary fibrosis was considered (Figure 2C). CT of the head was normal (Figure 3A), and the patient was conscious after withdrawal of the sedative. Although lung protective ventilation, recruitment maneuver and prone position ventilation were implemented, she did not tolerate attempts to wean from ECMO within 28 days of ECMO. She required continuous sedation and analgesia because of patient-ventilator asynchrony.

**Figure 4:**
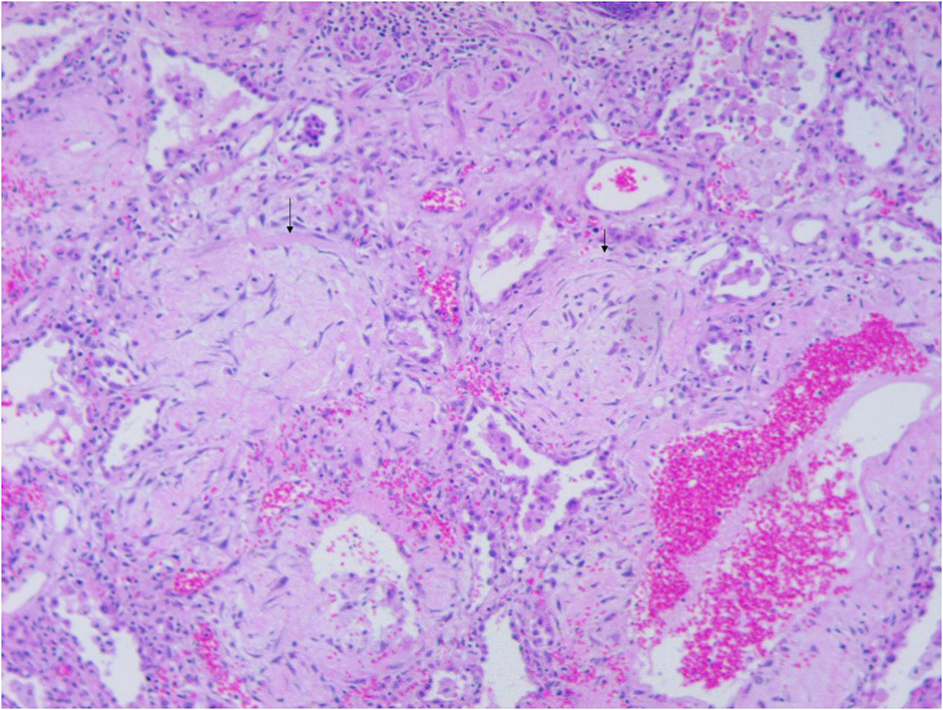
Microscopic examination of the explanted lung (hematoxylin-eosin stain, ×50) shows extensive consolidation of lung tissue and pulmonary interstitial fibrosis (arrowheads). Ring fibrosis connecting alveolar orifice rings and inflammatory cell infiltration into the alveolar walls with pneumocyte hyperplasia and squamous metaplasia.

Therefore, a decision was made to place the patient on the lung transplant waitlist, and she was subsequently transferred to the transplantation center for lung transplant evaluation. Anti-MDA5 antibody was tested by ELISA, and the result was positive. Based on these findings, the patient was diagnosed with CADM and ILD. At 31 days of ECMO, the patient underwent a successful sequential double lung transplant and received tacrolimus as an immunosuppressive regimen after the transplant. Her explant pathology showed extensive consolidation of lung tissue and pulmonary interstitial fibrosis (Figure 4). The patient's respiratory status gradually improved, and CT of the chest showed bilateral pulmonary scattered infiltration (Figure 2D), which was improved compared with previous imageological diagnosis. ECMO was weaned successfully 3 days after transplant, and the patient's oxygenation status did not deteriorate with ventilator support. The patient's state of consciousness deteriorated, and she presented with coma. Head CT showed bilateral parieto-occipital low-density lesions, which were considered to be due to PRES (Figure 3B). Since the condition was considered to be related to immunosuppressive agents, tacrolimus was suspended for 1 day, and the dosage was gradually reduced to 0.5 mg/day, after which the patient's consciousness returned. Unfortunately, the patient developed a disturbance of consciousness once more after hemodynamic instability, which may be related to implant infection; consciousness did not return after active treatment. After 14 days of lung transplant, the patient declined further treatment for financial reasons and was discharged.

## Discussion

CADM typically presents with characteristic cutaneous manifestations of classic dermatomyositis without muscle involvement; almost all patients with CADM present at least one characteristic skin lesion (1). In our present case, because of the absence of traditional muscle findings and atypical skin lesions after hospitalization, the diagnosis of CADM can be a challenge. In this case, facial erythema was noticed after induction of labor for a stillborn fetus, and the symptom vanished after oral prednisone administration. Meanwhile, the patient presented with no myalgia, and her creatine kinase level was normal during subsequent hospitalization. Unfortunately, this patient's symptoms were neglected in the local hospital for the first visit, leading to misdiagnosis. For pregnant and post-partum women, we may need to pay more attention to patients' autoimmune diseases, so as to provide patients with the proper diagnosis and treatment timely.

Patients with CADM are often combined with ILD. It is known that RP-ILD is more common in the CADM subset. Main common strategies for CADM include glucocorticoid pulse therapy and immunosuppressive therapy. Both drugs are often used together and considered to be valid. Furthermore, calcineurin inhibitors, plasma exchange, and hemoperfusion can be alternatives to these drugs when the combination therapy didn't work well. The patient initially presented with interstitial pneumonia and then developed refractory respiratory failure very quickly. Despite maximum respiratory support and the corresponding treatment, the patient's status was aggravated continuously with a life-threatening hypoxemia. The mechanism of onset of RP-ILD is ill-informed; reports have suggested that the condition is highly correlated with CADM and MDA5 antibody positivity (10). High SF has been shown to be another important indicator of poor prognosis of RP-ILD in CADM. The SF levels are reported to be higher with positive anti-MDA5 antibody than with negative anti-MDA5 antibody and higher in non-survivors than in survivors (11). Furthermore, anti-Ro-52 is also a risk factor for ILD in CADM (12). The patient experienced pneumomediastinum and pneumothorax with the development of CADM and RP-ILD. Pneumomediastinum has also been reported to be more common in CADM patients with positive anti-MDA5 antibody than in patients with negative anti-MDA5 antibody (13). These reports indicate that our patient had a high anti-MDA5 antibody titer, a high anti-Ro-52 antibody titer, a high ferritin level, and the complications of pneumomediastinum and pneumothorax, all of which indicated the diagnosis of clinically amyopathic dermatomyositis and showed a poor prognosis.

ECMO provides temporary cardiopulmonary support in patients with severe but potentially reversible cardiac and/or respiratory failure unresponsive to maximal conventional management. While it does not reverse the underlying lung disease, it acts as a bridge to recovery by offering patients more time for treatment therapies to take effect. It can also reduce ventilator-induced lung injury and oxygen toxicity caused by mechanical ventilation, which can add further damage to already damaged lungs. Last, chronic systemic disease or refractory end-stage pulmonary disease, which was resistant to conventional therapy, was considered a contraindication to ECMO in the past. Pulmonary interstitial fibrosis associated with RP-ILD in CADM is a rare indication for lung transplantation. However, ECMO can provide additional time for these patients who are being considered for lung transplant, and there are some reports of lung transplantation for RP-ILD (14). In the course of this patient, decreasing oxygenation index (PaO_2_/FiO_2_) and pneumomediastinum are clear indications of ECMO initiation in this patient, with oxygenation index showing collapsing pulmonary diffusion function and pneumomediastinum indicating the patient's own extreme breathing effort. On the other hand, ventilator maintenance of nearly 4 days prior to ECMO initiation avoids irreversible lung damage from prolonged mechanical ventilation.

Although after lung protective ventilation strategy, lung recruitment maneuver and prone position were all performed to support ECMO, pulmonary interstitial fibrosis gradually occurred, and the patient did not tolerate attempts to wean from ECMO. Our case indicates that ECMO has shown to be a valid rescue therapy in acute refractory respiratory failure secondary to RP-ILD and made further diagnosis and treatment possible. Although this patient did not completely recover her health in the end, ECMO added time to make an accurate diagnosis and offered the patient the opportunity for a lung transplantation.

Tacrolimus, a calcineurin inhibitor, is an effective immunosuppressive agent for the prevention of organ transplant rejection. Whereas, PRES is a rare and serious neurologic complication of tacrolimus. With binding to immunophilins to inhibit the calcineurin-mediated calcium-dependent signaling pathways, tacrolimus can activate T cells and IL-2. Meanwhile, calcineurin is also a mediator of neuronal function and drug toxicity is supposed to occur through impaired vasoconstriction of cerebrovascular vessels and dysregulation of the blood-brain barrier (9). Temporary cessation of tacrolimus and gradual reduction of the dosage may be effective strategies for managing PRES. However, these managements include risks of reducing immunosuppression, thereby leading to acute rejection. In our case, the strategy is to suspend tacrolimus for 1 day and gradually reduce the dosage to 0.5 mg/day. During the therapy, this patient's tacrolimus blood concentration was monitored once a day after lung transplantation and ranged from 2.7 to 18.6 ng/ml. The patient's neurological symptoms improved temporally, and signs of acute rejection were absent.

In conclusion, when patients experience RP-ILD for no apparent reason, CADM should be considered, especially in post-partum patients who are positive for anti-MDA5 and anti-Ro-52 antibody and have a high ferritin level and complications of pneumomediastinum and pneumothorax. ECMO should be considered as a supportive therapy and initiated early in patients in acute respiratory failure secondary to RP-ILD since it could provide a true opportunity to improve survival for such a rare disease and its potentially deadly complications. In cases of refractory respiratory failure and pulmonary fibrosis, lung transplant may be an option. PRES is not very common, but more attention should be paid to the patients who are using immunosuppression drugs. Our findings suggest that temporary cessation of tacrolimus and dosage reduction may be effective management strategies for PRES. The results of our case are frustrating, but more experience and further studies are needed to evaluate the true value of this method.

## Data Availability Statement

The original contributions presented in the study are included in the article/supplementary material, further inquiries can be directed to the corresponding authors.

## Ethics Statement

Written informed consent was obtained from the individual(s) for the publication of any potentially identifiable images or data included in this article.

## Author Contributions

All authors listed have made a substantial, direct and intellectual contribution to the work, and approved it for publication.

## Conflict of Interest

The authors declare that the research was conducted in the absence of any commercial or financial relationships that could be construed as a potential conflict of interest.

## Publisher's Note

All claims expressed in this article are solely those of the authors and do not necessarily represent those of their affiliated organizations, or those of the publisher, the editors and the reviewers. Any product that may be evaluated in this article, or claim that may be made by its manufacturer, is not guaranteed or endorsed by the publisher.
